# The simulation of stress fibre and focal adhesion development in cells on patterned substrates

**DOI:** 10.1098/rsif.2007.1182

**Published:** 2007-10-16

**Authors:** Amit Pathak, Vikram S Deshpande, Robert M McMeeking, Anthony G Evans

**Affiliations:** 1 Department of Mechanical Engineering, University of CaliforniaSanta Barbara, CA 93106, USA; 2 Department of Materials, University of CaliforniaSanta Barbara, CA 93106, USA

**Keywords:** mechano-sensitivity, stress fibre, actin, micro-pattern

## Abstract

The remodelling of the cytoskeleton and focal adhesion (FA) distributions for cells on substrates with micro-patterned ligand patches is investigated using a bio-chemo-mechanical model. We investigate the effect of ligand pattern shape on the cytoskeletal arrangements and FA distributions for cells having approximately the same area. The cytoskeleton model accounts for the dynamic rearrangement of the actin/myosin stress fibres. It entails the highly nonlinear interactions between signalling, the kinetics of tension-dependent stress-fibre formation/dissolution and stress-dependent contractility. This model is coupled with another model that governs FA formation and accounts for the mechano-sensitivity of the adhesions from thermodynamic considerations. This coupled modelling scheme is shown to capture a variety of key experimental observations including: (i) the formation of high concentrations of stress fibres and FAs at the periphery of circular and triangular, convex-shaped ligand patterns; (ii) the development of high FA concentrations along the edges of the V-, T-, Y- and U-shaped concave ligand patterns; and (iii) the formation of highly aligned stress fibres along the non-adhered edges of cells on the concave ligand patterns. When appropriately calibrated, the model also accurately predicts the radii of curvature of the non-adhered edges of cells on the concave-shaped ligand patterns.

## 1. Introduction

Cell adhesion to the extracellular matrix (ECM) is a crucial phenomenon in many cellular functions, ranging from migration and proliferation to apoptosis (Boudreau & Bissell 1998; Schwartz & Ginsberg 2002). The focal adhesions (FAs) are the primary subcellular structures that mediate the regulatory effects of ECM adhesion on cell behaviour. These macromolecular complexes mediate cell anchorage to ECM by physically coupling integrins to the contractile actin cytoskeleton (Wang *et al*. 1993; Sastry & Burridge 2000); they also orient and concentrate signalling proteins at sites of integrin binding and clustering (Sastry & Burridge 2000; Alenghat & Ingber 2002). The FAs function not only as the mechanical linkages that anchor the intracellular cytoskeleton to bound integrins (and the ECM), but they also serve as biochemical signalling hubs for many regulatory pathways; regulation of the assembly and disassembly of the FAs is critical for controlling cell function (Wang *et al*. 1993; Schwartz & Ginsberg 2002).

Early studies of cell adhesion on flat substrates described several distinct processes, including the initial clustering of integrins when they bind to ECM ligands, the associated formation of FAs and progressive maturation of these adhesion complexes as cells undergo active spreading and flattening. Because these substrates provide homogeneous unlimited adhesion, the cell migrates and the cytoskeleton remodels continuously, with consequent loss of the reproducibility of cell shape and cytoskeleton organization operable in tissues. By confining cells on micro-patterns, this movement is impeded and a reproducible shape imposed (Singhvi *et al*. 1994). Consequently, fully adhesive or convex-shaped micro-patterns have been used to modulate cytoskeleton organization and FA distributions (Parker *et al*. 2002; Chen *et al*. 2003) with the following two outcomes: (i) most of the cells tend to organize long stress fibres, with FAs concentrating at the cell periphery, especially in high-curvature regions and (ii) the rate of growth of the FAs is proportional to the local tractions, with the adhesions disintegrating when cell contractility is curtailed by adding reagents such as 2,3-butanedione monoxime (BDM) to the cell culture (Chen *et al*. 2003). External force applied to the cytoskeleton through the cell membrane also stimulates FA growth when cell contractility has been downregulated by BDM (Riveline *et al*. 2001). Furthermore, in both the active and the BDM-downregulated states, FA size is proportional to the force (Balaban *et al*. 2001). These and other aspects of mechano-sensitivity of FA complexes have been summarized by Bershadsky *et al*. (2003, 2006).

By decoupling cell and ligand patch shapes for both convex and concave ligand patterns, while retaining constant cell area, Théry *et al*. (2006) reproduced, *in vitro*, the heterogeneity of the chemo-mechanical environment that cells encounter *in vivo*. For cells on concave V-, T-, Y- and U-shaped ligand patterns, they measured the steady-state stress-fibre and FA concentrations, as parametrized by the actin and vinculin distributions (figure 1). These images reveal that: (i) high FA concentrations form along the periphery of ligand patches (rather than at the cell periphery) and (ii) on a concave-shaped patch, the stress-fibre concentrations are the highest along the non-adhered edge.

Prior modelling schemes that attempt to duplicate these phenomena have had limitations: (i) the dynamics inherent in the contractility of the stress fibres (causing them to ‘strengthen’ as the adhesion stiffness increases) has not been incorporated (Novak *et al*. 2004; Shemesh *et al*. 2005), (ii) the influence of the traction forces on FA assembly at the contact sites has not been analysed within a complete mechanical context (Novak *et al*. 2004), and (iii) the dynamic rearrangement of the cytoskeleton in response to external chemical and mechanical cues has not been incorporated in an overall constitutive framework for the stress-fibre and FA dynamics. Aspects of these limitations have been addressed in models that predict the stabilization of focal complexes, and FA growth, in response to either a force or an imposed strain (Nicolas *et al*. 2004; Bruinsma 2005; Besser & Safran 2006; Nicolas & Safran 2006). This has been achieved by incorporating the activated state of FA proteins, thereby enabling self-assembly. A more recent augmentation (Deshpande *et al*. in press) describes a coupled thermodynamic and mechanical framework that connects stress-fibre formation kinetics, their contractility and the force sensitivity of FA formation and growth. One aspect characterizes the dynamic contractile actin/myosin stress-fibre machinery governing stress generation inside the cell. Another is based on thermodynamic equilibrium of the integrins in their low- and high-affinity states; see Lodish *et al*. (1995) for a discussion on the states of the integrin molecules. When coupled, the model simultaneously characterizes the development of the FAs and the stress fibres. This bio-chemo-mechanical modelling approach not only incorporates the fundamental biological phenomena, but also appears to be consistent with experimental observations including: (i) the accumulation of FAs at the cell periphery (Deshpande *et al*. in press) for a *one-dimensional cell*, (ii) the high density of stress fibres at the attachments to the ECM as well as at locations where external forces are imposed (Deshpande *et al*. 2007), and (iii) the scaling of the forces generated by the cell with the substrate stiffness (Deshpande *et al*. 2006) and (for a cell on a bed of micro-needles) the scaling with the number of posts (Deshpande *et al*. 2007). The model also predicts the effect of cyclic straining of the substrate on the stress-fibre orientations (Wei *et al*. in press). Nevertheless, multiple verification tests are required to establish the fidelity and versatility of this model before it can serve as a comprehensive and robust tool for broadly simulating cellular responses.

The intent of this article is twofold. We extend the one-dimensional model in Deshpande *et al*. (in press) to two-dimensional situations so that we can simulate the behaviour of cells adhered to flat substrates. Thereafter, we make a significant step towards validation by carrying out such two-dimensional simulations and then comparing predictions of the actin and vinculin distributions, as well as the curvatures of the non-adhered cell edges, with the aforementioned observations and measurements (Théry *et al*. 2006).

## 2. Synopsis of the bio-chemo-mechanical model

We envisage a two-dimensional cell, thickness *b* lying in the *x*_1_−*x*_2_ plane on a substrate having its normal along the *x*_3_-direction (figure 2). We describe the governing equations for the coupled contractile and adhesive response using Cartesian tensor notation. The interaction of the cell with a substrate incorporates two components that link in a highly nonlinear manner. (i) Following an activation signal, the model calculates the development of the stress fibres subject to forces or displacements imposed along the membrane, at the interface between the cell and its substrate. The outputs are the spatial and temporal (time *t*) distributions of the stress-fibre concentration, *η*(*x*_*i*_, *ϕ*, *t*) (see figure 2 for the definition of the angle *ϕ*) and the stresses $\sum_{ij}\left(x_{i},t\right)$ generated by the resulting stress-fibre contractility. (ii) These stresses apply tractions to the attached FAs and, thereby, control their spatial and temporal developments, as parametrized through the high-affinity integrin concentration, *ξ*_H_(*x*_*i*_, *t*) along the cell membrane. In turn, the forces generated by the distortion of the FAs balance those generated by the stress fibres. Thus, the formation of stress fibres is coupled to their contractile action on the (mechano-sensitive) integrins that, in turn, generates and grows focal adhesions at the terminations of the acto-myosin strands. We shall show that, since *force equilibrium dictates* that the stress fibres apply the greatest force at the perimeter of the adhered portion of the cell membrane, the FAs likewise grow to their greatest extent at the perimeter.

The mechanical equilibrium equations coupling the stresses in the cell with the forces *F*_*i*_ per high-affinity integrin in the FAs are written as

(2.1a) $$b\left(\frac{\partial \Sigma_{11}}{\partial x_{1}}+\frac{\partial \Sigma_{12}}{\partial x_{2}}\right)=\xi_{\text{H}}F_{1}\;\text{and}$$

(2.1b) $$b\left(\frac{\partial \Sigma_{12}}{\partial x_{1}}+\frac{\partial \Sigma_{22}}{\partial x_{2}}\right)=\xi_{\text{H}}F_{2},$$

where *x*_*i*_ is the coordinate of a material point of the cell in the current configuration (note that here *F*_*i*_ is the force exerted by the cell on the integrin–ligand complex). In the remainder of this section, the two components of the model are described. The manifestation of this interaction within actual cells is elaborated in §5. For a detailed justification of the model and its relationship to the underlying biochemistry, we refer the reader to our previous paper, Deshpande *et al*. (2007), and its on-line supporting material.

### 2.1 Stress-fibre contractility model

A recent bio-chemo-mechanical model captures the formation and dissociation of stress fibres, as well as the associated generation of tension and contractility (Deshpande *et al*. 2006, 2007). The model differs from the purely passive viscoelastic models in the prior literature by virtue of its incorporation of contractility, through the Hill-like law (Hill 1938) described below. In the model, actin polymerization (leading to stress-fibre formation in the cell) is governed by three coupled phenomena: (i) an activation signal, (ii) tension-dependent stress-fibre kinetics, and (iii) a force generation mechanism governed by cross-bridge cycling between actin and myosin filaments.

#### 2.1.1 Phenomenon I

Stress-fibre formation is initiated by a biochemical or mechanical perturbation that triggers a signalling cascade within the cell. We model this signal as an externally initiated, exponentially decaying pulse having level *C* given by

(2.2) $$C=\text{exp}(-t_{\text{I}}/\theta ),$$

where *θ* is a constant that controls the decay rate of the signal and *t*_I_ is the time measured from the instant the most recent signal is imposed. In reality, the clustering of high-affinity integrins in FAs (due to either the application of mechanical forces or the presence of appropriate ligand molecules) internally initiates the signalling cascade that results in the formation of stress fibres.^1^ The signal subsides due to restoring mechanisms that include: (i) the dephosphorylation of IP_3_ by specific phosphatases to form IP_2_ and IP_4_ which closes the Ca^2+^ gates on the membrane of the endoplasmic reticulum (ER) and (ii) pumping of Ca^2+^ from the cytosol into the ER, both augmented by further complexities in the reactions of the G-proteins involved in the signalling cascade. The complex signalling pathways that govern the rise and fall of this activation signal are neglected in this analysis, and we model the activation process due to the contact of the cell with the ligand pattern on the substrate through a pre-specified activation signal of the form given by equation (2.2). It is worth noting here that signal transduction within the cell follows the interconnected pathway of integrins, cytoskeleton filaments and nucleus (Maniotis *et al*. 1997), which in turn affects the mechanical and biochemical responses of nucleus—such effects are neglected in the present model.

#### 2.1.2 *Phenomenon II*

Following the activation signal, stress fibres form with a local concentration parametrized by an activation level, *η* (0≤*η*≤1), a quantity representing the ratio of the polymerized actin and phosphorylated myosin concentrations within a stress-fibre bundle to the maximum concentrations permitted by the biochemistry. Stress fibres are allowed to form in any direction, with degree of polymerization at an angle *ϕ* with respect to the *x*_1_-axis (figure 2) denoted by *η*(*x*_*i*_, *ϕ*, *t*). The rate of formation of the stress fibres is governed by a kinetic law incorporating forward and backward rates having the form

(2.3) $$\dot{\eta }(x_{i},\phi ,t)=[1-\eta (x_{i},\phi ,t)]\frac{C{\bar{k}}_{\text{f}}}{\theta }-\left(1-\frac{\sigma (x_{i},\phi ,t)}{\sigma_{0}(x_{i},\phi ,t)}\right)\eta (x_{i},\phi ,t)\frac{{\bar{k}}_{\text{b}}}{\theta },$$

where the overdot denotes differentiation with respect to time *t* measured from the instant of the first signal; *σ*(*x*_*i*_, *ϕ*, *t*) is the tension in the stress fibre at position *x*_*i*_ and time *t* formed in the direction *ϕ*; and *σ*_0_≡*ησ*_max_ is the corresponding isometric tension at activation level *η* with *σ*_max_ being the tensile stress at full activation (*η*=1). Note that equation (2.3) embodies standard (linear) kinetics for the polymerization of the stress fibres (the first term on the r.h.s.) and their depolymerization (the second term on the r.h.s.) with the following features: (i) the forward (polymerization) reaction rate is proportional to the signal, (ii) shortly after its initiation, the reaction rate is high, and (iii) after the signal decays, the reaction rate slows due to lack of stimulation. The kinetics of the depolymerization is affected by the tension in the stress fibres. We surmise that the fibres are stable under isometric tension. Thus their dissociation is suppressed whenever *σ*=*σ*_0_, consistent with the observation that the tension generated in the stress fibres arises from the same source as their stability against dissociation: through the cross-bridging dynamics of the myosin molecular motors that hold the acto-myosin strands together (Deshpande *et al*. 2006). The implications are as follows: (i) an isometric condition in a stress fibre shuts off the depolymerization reaction completely and (ii) when the tension falls below this level (due to shrinkage of the acto-myosin strand), depolymerization can proceed, owing to the diminished likelihood that a myosin head engages a connection point in the actin strand. Such a reduction is also the source of the lower tension in the stress fibre. Note that the dimensionless constants ${\bar{k}}_{\text{f}}$ and ${\bar{k}}_{\text{b}}$ in equation (2.3) govern the rates of formation and dissociation, respectively, of the fibres.

#### 2.1.3 Phenomenon III

In turn, the stress *σ* induced in the fibres is related to the fibre contraction/extension rate $\dot{\epsilon }$ (the rate at which deformation takes place in the stress fibre) by virtue of the cross-bridge cycling between the actin and myosin filaments. The contractility is spontaneous and driven by the cross-bridge cycling of myosin motors between actin fibres, and is present in every stress fibre in the model. Thus, as soon as a stress fibre is formed by activation, contractile tension is generated by the myosin motors. Furthermore, and as noted above, if the stress fibre is held in an isometric condition, the tension generated by the cross-bridge cycling is the greatest, as in the Hill–Huxley (Hill 1938; Huxley 1957) dynamics of acto-myosin strands. In contrast, if the stress fibre is allowed to shrink, due to the action of the cross-bridging, the tension it generates falls, due to the reduced likelihood of the myosin heads engaging connection points on the actin fibres. Thus, a version of the Hill-like (Hill 1938) contractility law is employed to model these dynamics and specified as

(2.4) $$\frac{\sigma }{\sigma_{0}}=\{\begin{array}{ll} 0 & \frac{\dot{\epsilon }}{{\dot{\epsilon }}_{0}}<-\frac{\eta }{{\bar{k}}_{\text{v}}}\\ 1+\frac{{\bar{k}}_{\text{v}}}{\eta }\left(\frac{\dot{\epsilon }}{{\dot{\epsilon }}_{0}}\right) & -\frac{\eta }{{\bar{k}}_{\text{v}}}\leq \frac{\dot{\epsilon }}{{\dot{\epsilon }}_{0}}\leq 0\\ 1 & \frac{\dot{\epsilon }}{{\dot{\epsilon }}_{0}}>0 \end{array},$$

where the rate sensitivity coefficient, ${\bar{k}}_{\text{v}}$, is the reduction in fibre stress upon increasing the shortening rate by ${\dot{\epsilon }}_{0}$, where the tension reduction is expressed as a fraction of *σ*_max_. Note that this constitutive law is local in nature. Namely, the stress at a given point depends only on the strain rate at that point. Similarly, the coupling between the activation and the stress in equation (2.3) is local. The condition that mechanical equilibrium be satisfied, embodied in equations (2.1*a*) and (2.1*b*), imposes constraints on the spatial gradients of the stress tensor. Consequently, the correlation among neighbouring points arises from the nature of the strain rate and mechanical equilibrium.

The above phenomena, taken together, clarify that the activation signal, leading to formation of stress fibres, gives rise directly to forces (generated by cross-bridge cycling) that are, in turn, imposed on the FAs attached to the acto-myosin strands.

In a two-dimensional setting, at angle *ϕ*, the axial fibre strain rate $\dot{\epsilon }$ (representing the rate at which the stress fibres deform) is related to the material strain rate ${\dot{\epsilon }}_{ij}$ by

(2.5) $$\dot{\epsilon }\equiv {\dot{\epsilon }}_{11}\;{\text{cos}}^{2}\phi +{\dot{\epsilon }}_{22}\;{\text{sin}}^{2}\phi +{\dot{\epsilon }}_{12}\;\text{sin}\;2\phi$$

and the average stress generated by the fibres follows from a homogenization analysis as

(2.6) $$S_{ij}(x_{i},\phi ,t)=\frac{1}{\pi }\int_{-\pi /2}^{\pi /2}\left(\begin{array}{cc} \sigma (x_{i},\phi ,t){\text{cos}}^{2}\phi & \frac{\sigma (x_{i},\phi ,t)}{2}\text{sin}\;2\phi \\ \frac{\sigma (x_{i},\phi ,t)}{2}\text{sin}\;2\phi & \sigma (x_{i},\phi ,t)\;{\text{sin}}^{2}\;\phi \end{array}\right)\text{d}\phi .$$

The constitutive description for the cell is completed by including contributions from passive elasticity, attributed to intermediate filaments and microtubules of the cytoskeleton attached to the nuclear and plasma membranes. These act in parallel with the active elements, whereupon additive decomposition gives the total stress as

(2.7) $$\Sigma_{ij}=S_{ij}+\left(\frac{E\nu }{\left(1-2\nu )(1+\nu \right)}\epsilon_{kk}\delta_{ij}+\frac{E}{\left(1+\nu \right)}\epsilon_{ij}\right),$$

where *δ*_*ij*_ is the Kronecker delta and (for a linear response) *E* is Young's modulus and *ν* is the Poisson ratio. The above equations are valid in a small or infinitesimal deformation setting; readers are referred to Deshpande *et al*. (2007) for the finite strain and three-dimensional generalization.

In summary, this component of the model allows calculation of the spatial and temporal variations in the stresses *Σ*_*ij*_ in the cell as well as the local concentration *η*(*x*_*i*_, *ϕ*, *t*) of the stress fibres along the direction *ϕ* at position *x*_*i*_ in the cell at time *t*.

### 2.2 FA model

A viable model for the formation and growth of FAs must account for the cooperativity between the adhesions and foregoing cell contractility. This has been accomplished in a recent thermodynamically motivated model by Deshpande *et al*. (in press), predicated upon the observation that any physio-chemical system evolves such that its free energy reduces and homogenizes: a phenomenon that occurs whether the system conserves or dissipates energy (Gaskell 1973).

The Deshpande *et al*. (in press) model relies on the existence in two conformational states for the integrins: low and high affinities. Only the high-affinity integrins interact with the ligand molecules on the ECM to form complexes. The low-affinity integrins remain unbonded. Since the formation (or dissociation) of the FAs depends on the relative stabilities of the high- and low-affinity integrins, an examination of the chemical potentials of these two states becomes a prerequisite to the prediction of FA formation. We commence from a thermodynamic baseline that the ‘straight’ state of the integrins is less stable than the ‘bent’ or low-affinity state (McCleverty & Liddington 2003) suggesting that the high-affinity state has the higher reference chemical potential (*μ*_H_>*μ*_L_).

The chemical potential of the low-affinity integrins at concentration *ξ*_L_ is dependent on their internal energy and configurational entropy in accordance with

(2.8) $${Χ}_{\text{L}}=\mu_{\text{L}}+kT\;\text{ln}(\xi_{\text{L}}/\xi_{\text{R}}),$$

where *μ*_L_ is their reference chemical potential; *ξ*_R_ their reference binder concentration; *k* the Boltzmann constant; and *T* the absolute temperature. The second term in equation (2.8) is a standard expression for the configurational entropy (Gaskell 1973) in the limit of a dilute binder concentration.

For geometrical reasons, the straight architecture of the high-affinity integrins permits the interaction of its receptor with the ligand molecules on the ECM. Thus, the high-affinity integrins have additional contributions to their chemical potential. These contributions involve the potential energy stored due to the stretching of the integrin–ligand complexes and a term related to the mechanical work done by the stress fibres. The ensuing potential is

(2.9) $${Χ}_{\text{H}}=\mu_{\text{H}}+kT\;\text{ln}(\xi_{\text{H}}/\xi_{\text{R}})+\Phi (\Delta_{i})-F_{i}\Delta_{i},$$

where *Φ*(*Δ*_*i*_) is the stretch energy stored in the integrin–ligand complex. The −*F*_*i*_*Δ*_*i*_ term in (2.9) is the mechanical work term that represents the loss in free energy due to the stretch *Δ*_*i*_ of the integrin–ligand complex by the force *F*_*i*_. (It is analogous to the pressure–volume term in the thermodynamics of gases.) In molecular terms this implies that the stretch of the ligand–integrin complex can stabilize the adhesion by lowering the free energy *Χ*_H_. Formally, the energy *Φ* and force *F*_*i*_ are not independent; rather the work-conjugate force, *F*_*i*_, is related to the stretch *Δ*_*i*_ via the relation

(2.10) $$F_{i}\equiv \frac{\partial \Phi }{\partial \Delta_{i}}.$$

The formulae (2.8)–(2.10) are used widely to characterize the thermodynamic states of solids (Gaskell 1973). The work term in (2.9) had been identified previously (Shemesh *et al*. 2005) as an important constituent of the thermodynamic state of the FAs. However, the terms due to the stored energy and the configurational entropy have been neglected in these prior assessments. Yet they are critical to the formulation of a general framework that explicitly accounts for the cytoskeletal response, inclusive of remodelling as a result of contractility, spreading, etc.

Gradients in the foregoing chemical potentials motivate the fluxes of the integrins. Two kinetic processes involved (Deshpande *et al*. in press) are as follows:

- those governing the rate of conversion of the low-affinity integrins to their high-affinity state (and vice versa) and
- diffusive fluxes of the low-affinity integrins along the plasma membrane.

These kinetics are typically fast compared with all other time scales involved (Deshpande *et al*. in press). Consequently, in this simple treatment we neglect the diffusion of the low-affinity integrins and the kinetics between the low- and high-affinity states of the integrins at any location on the plasma membrane. These simplifying assumptions imply that the concentrations are given by thermodynamic equilibrium between the low- and high-affinity binders, i.e. at each location *x*_*i*_ on the plasma membrane

(2.11) $${Χ}_{\text{H}}={Χ}_{\text{L}},$$

which specifies that the high- and low-affinity integrin concentrations are related to the force *F*_*i*_ via

(2.12a) $$\xi_{\text{H}}=\frac{\xi_{0}}{\text{exp}\left[\frac{\mu_{\text{H}}-\mu_{\text{L}}+\Phi -F_{i}\Delta_{i}}{kT}\right]+1}\;\text{and}$$

(2.12b) $$\xi_{\text{L}}=\frac{\xi_{0}}{\text{exp}\left[-\frac{\mu_{\text{H}}-\mu_{\text{L}}+\Phi -F_{i}\Delta_{i}}{kT}\right]+1},$$

respectively, where *ξ*_0_=*ξ*_H_+*ξ*_L_ is the total concentration of the high- and low-affinity binders at the location *x*_*i*_. It is evident that with decreasing *Φ*−*F*_*i*_*Δ*_*i*_ (which typically occurs when the tensile force *F*_*i*_ increases), the concentration *ξ*_H_ of the high-affinity integrins increases due to the conversion of the low-affinity integrins to their high-affinity state, i.e. *ξ*_L_ simultaneously reduces.

These are the governing equations for the integrin concentrations along the plasma membrane. As noted above, they couple with the contractility of the cell through the equilibrium relation, equations (2.1*a*) and (2.1*b*). To complete the thermodynamic representation it remains to specify the form of the energy *Φ*(*Δ*_*i*_) and the associated force *F*_*i*_ in the integrin–ligand complex. Rather than employing a complex interaction, such as the Lennard-Jones potential (Lennard-Jones 1931), we use the simplest functional form. This is a piecewise quadratic potential expressed as (Deshpande *et al*. in press)

(2.13) $$\Phi =\{\begin{array}{ll} (1/2)\kappa_{\text{s}}\Delta_{\text{e}}^{2} & \Delta_{\text{e}}\leq \Delta_{n}\\ -\kappa_{\text{s}}\Delta_{n}^{2}+2\kappa_{\text{s}}\Delta_{n}\Delta_{\text{e}}-(1/2)\kappa_{\text{s}}\Delta_{\text{e}}^{2} & \Delta_{n}<\Delta_{\text{e}}\leq 2\Delta_{n}\\ \kappa_{\text{s}}\Delta_{n}^{2} & \Delta_{\text{e}}>2\Delta_{n} \end{array},$$

where $\gamma \equiv \Phi (\Delta_{i}\to \infty )=\kappa_{\text{s}}\Delta_{n}^{2}$ is the surface energy of the high-affinity integrins; the effective stretch $\Delta_{\text{e}}\equiv \sqrt{\Delta_{1}^{2}+\Delta_{2}^{2}}$; and *κ*_s_ is the stiffness of the integrin–ligand complex. The maximum force, *κ*_s_*Δ*_*n*_, occurs at a stretch *Δ*_e_=*Δ*_*n*_.

We relate the evolution of the stretch *Δ*_*i*_ to the displacement *u*_*i*_ of the material point on the cell membrane in contact with the ligand patch on the substrate as (Deshpande *et al*. in press)

(2.14) $${\dot{\Delta }}_{i}=\{\begin{array}{ll} {\dot{u}}_{i} & \Delta_{\text{e}}\leq \Delta_{n}\;\text{or}\;\left[\frac{\partial \Phi }{\partial \Delta_{\text{e}}}{\dot{\Delta }}_{\text{e}}<0\right]\\ 0 & \text{otherwise} \end{array},$$

where we assume a rigid substrate (see Deshpande *et al*. (in press) for the generalization to a deformable substrate).

Those integrins not in contact with the ligand patch are assumed to be isolated. Namely, they are unable to interact with any ligand molecules. Accordingly, we assume that these integrins are unbonded with *Δ*_*i*_→∞ such that the interaction force *F*_*i*_=0 and energy *Φ*=*γ*. This implies that on portions of the cell membrane not in contact with the ligand patch, integrins have concentrations given by

(2.15a) $$\xi_{\text{H}}=\frac{\xi_{0}}{\text{exp}\left[\frac{\mu_{\text{H}}-\mu_{\text{L}}+\gamma }{kT}\right]+1}\;\text{and}$$

(2.15b) $$\xi_{\text{L}}=\frac{\xi_{0}}{\text{exp}\left[-\frac{\mu_{\text{H}}-\mu_{\text{L}}+\gamma }{kT}\right]+1}.$$

The integrin concentrations vary between those in (2.12*a*) and (2.12*b*) to those in (2.15*a*) and (2.15*b*) over a transition zone of size $\approx \Delta_{n}$ which is of the order of nanometres. Given that cell sizes are in the micrometre range we neglect the details of this transition zone. Moreover, we neglect the change in the integrin concentration due to the changes in the area of the cell. This change could be accounted for by replacing the numerator in equations (2.12*a*), (2.12*b*), (2.15*a*) and (2.15*b*) by *ξ*_0_(*ϵ*_11_+*ϵ*_22_). However, the strains in the examples presented subsequently are sufficiently small that neglecting the contribution (*ϵ*_11_+*ϵ*_22_) is deemed acceptable.

In summary, the tendency of the system to reduce and homogenize its free energy enables the coupled model to determine the following: (i) the concentration of integrins on the membrane, (ii) the separation of integrins into families of bent and straight conformations, and (iii) the distribution of stress fibres in the cytoskeleton. The associated thermodynamics do not imply that the cell is undergoing equilibrium processes. Rather, the lowering of the free energy is qualified by the kinetics of the dissipative, non-equilibrium processes taking place. Such an approach has been applied successfully to simulate a variety of non-equilibrium physical and chemical phenomena, such as the creep deformation of metals with mass transport-driven cavitation (Needleman & Rice 1980), sintering (Svoboda & Riedel 1995; Sun *et al*. 1996; Parhami *et al*. 1999; Redanz & McMeeking 2003), grain growth (Cocks & Gill 1996; Gill & Cocks 1996; Du *et al*. 2003) and oxidation (Suo *et al*. 2003). Within this general framework, to facilitate the modelling, the various processes are incorporated using different strategies. (a) In some, the kinetic evolution laws operate implicitly to minimize the system free energy, such as the formation and dissolution of the stress fibres and their myosin-driven contractility. (b) Others are introduced by means of an explicit free energy minimization coupled to a kinetic law controlling the process, such as the motion of the bent, low-affinity integrins on the membrane. (c) Yet others are identified by an equilibrium statement based on the associated kinetics which being much faster than those for the rate-limiting behaviour of the cell: (i) the partition of integrins on the membrane into a high affinity, straight family and a low affinity, bent set and (ii) the binding and unbinding of stress fibres with integrins to form the FA as well as the integrins with ligands on the substrate.

Note that Bischofs & Schwarz (2003) and Bischofs *et al*. (2004) have taken an approach to explain the cytoskeletal and focal adhesive organizations of cells by deducing that they minimize the work expended to achieve a critical level of force that they apply to a substrate. This model successfully explains the differing response of cells to compliant versus stiff substrates and a variety of stiffness-modulating features of surfaces to which they attach. The new model of Deshpande *et al*. (in press) builds on this concept of energy minimization, extending it to embrace the principle of reduction of free energy as the driving force for the processes involved. Future work will investigate the extent of consistency between the approach of Deshpande *et al*. (in press) and that of Bischofs & Schwarz (2003) and Bischofs *et al*. (2004).

### 2.3 Finite element implementation

The models for the cytoskeletal contractility and FAs have been implemented in the commercial finite element (FE) code ABAQUS, Inc. (2004). The contractility model is implemented as a user-defined material (Deshpande *et al*. 2006, 2007). In Deshpande *et al*. (in press) only a one-dimensional version of the adhesion model was investigated. Here we solve the above described two-dimensional thermomechanical partial differential equations for cell adhesion using the FE method. Specifically, FAs were included in the model by employing the user-defined interface option in ABAQUS. In the ABAQUS surface interaction terminology we treat that portion of the substrate with the ligand patch as the *master* surface, whereas the *slave* surface is the entirety of the cell in contact with the substrate. Thus, FAs are allowed to form only where the ligand patch is present.

The cells were modelled using deformable four-noded membrane elements (M3D4 in the ABAQUS terminology) and the response solved for in a finite strain setting (i.e. the effects of geometry changes on the momentum balance and rigid body rotations are taken into account). Typical FE meshes comprised elements of average size 0.25 μm (for cells with a leading dimension of approx. 46 μm) with the meshes refined near the edges of the ligand patches to capture large parameter gradients. We conducted mesh sensitivity studies and confirmed that reducing the mesh size below 0.25 μm did not change the results appreciably.

## 3. Correlation between model parameters and experimental images

FAs are imaged in experiments by staining for the protein vinculin, which directly correlates to the concentration of the high-affinity integrins (or *ξ*_H_ in the model). Consequently, we seek a correspondence between the normalized quantity *ξ*_H_/*ξ*_0_ in the model and the vinculin distribution in the experiments. The corresponding characterizing parameter for the *actin* (or stress fibre) distributions is not chosen so straightforwardly. Most techniques only image the dominant stress fibres. The very fine mesh-work of actin filaments is not visible when standard epifluorescence or confocal microscopes are used. Thus, to correlate the observations with the predictions we define a circular variance that provides an estimate of how tightly the stress fibres are clustered around a particular orientation *ϕ*:

(3.1) $$\Gamma \equiv 1-\frac{\bar{\eta }}{\eta_{\text{max}}}.$$

Here *η*_max_ is the maximum polymerization level which occurs at orientation *ϕ*_s_ while $\bar{\eta }$ is an average value defined as $\bar{\eta }\equiv 1/\pi \int_{-\pi /2}^{\pi /2}\eta \;\text{d}\phi$. The value of *Γ* varies from 0 to 1, corresponding to perfectly uniform and totally aligned distributions, respectively. Subsequently, we show that the distributions of *Γ* predicted by the model consistently correspond with the experimental actin images. In all ensuing plots, we include line segments illustrating the local orientation *ϕ*_s_ with the length of the line segments proportional to the local value of *Γ*.

## 4. Choice of material parameters

Unless otherwise specified, the following reference material parameters are used, with temperature *T*=310 K. The decay constant of the signal is *θ*=720 s, the passive Young's modulus is *E*=0.08 kPa and the Poisson ratio *ν*=0.3.^2^ The non-dimensional reaction rate constants are ${\bar{k}}_{\text{f}}=10$ and ${\bar{k}}_{\text{b}}=1.0$, while the maximum tension exerted by the stress fibres is *σ*_max_=4.0 kPa. Independent assessments conducted in the context of cells on a bed of micro-needles (McGarry *et al*. submitted) suggest that ${\bar{k}}_{\text{v}}=6$ with ${\dot{\epsilon }}_{0}=2.8\times 10^{-4}\;{\text{s}}^{-1}$. Thus, with the exception of ${\bar{k}}_{\text{v}}$ all parameters are the same as those used previously (Deshpande *et al*. 2006, 2007). Many of the parameters have been chosen to give acceptable correspondence with experiments carried out by Tan *et al*. (2003). For example, the signal decay time, *θ*=720 s, was selected to ensure that the measured time history of forces applied by the cell was realistically simulated. Moreover, many phenomena observed in experiments have been simulated successfully when the preceding values are used: (i) the scaling of the forces generated by the cell with substrate stiffness (Deshpande *et al*. 2006), (ii) the scaling with the number of underlying posts of the forces generated by a cell adhered to micro-needles (Deshpande *et al*. 2007), and (iii) the effect of cyclic straining of a substrate on the stress-fibre orientations in the cytoskeleton (Wei *et al*. in press).

The FA properties were chosen in two steps. (i) The parameters were constrained to lie within commonly accepted ranges. (ii) Specific values were tuned to obtain broad agreement with the observations in Théry *et al*. (2006). The total concentration of the high- and low-affinity binders *ξ*_0_ is taken to be 1000 integrins μm^−2^ (Lauffenburger & Linderman 1996). The difference in the reference chemical potentials of the high- and low-affinity integrins is taken as *μ*_H_−*μ*_L_=5*kT*, such that the ratio *ξ*_L_/*ξ*_H_≈150 for a cell with contractility curtailed (*F*_*i*_=*Φ*=0) is consistent with observations (McCleverty & Liddington 2003). Measurements suggest that the strength of the integrin–ligand complex varies in the range 2→10 pN (Merkel *et al*. 1999; Rinko *et al*. 2004), while the surface energy per integrin is in the range 5→50*kT* (Leckband & Israelachvili 2001). To be consistent, we choose an integrin–ligand complex stiffness, *κ*_s_=0.015 nN μm^−1^ and value of the stretch at maximum force, *Δ*_*n*_=130 nm, giving a strength approximately 2 pN and a surface energy *γ*∼60*kT*, slightly higher than suggested elsewhere (Leckband & Israelachvili 2001).

The choice of these parameters is expected to be sensitive to cell type. For example, myocytes and fibroblasts are capable of exerting much larger contractile forces than the retinal endothelial cells employed by Théry *et al*. (2006). Thus, while modelling the contractility of fibroblast on a bed of micro-needles, McGarry *et al*. (submitted) used *σ*_max_=20 kPa in order to obtain good agreement with force measurements (Tan *et al*. 2003).

## 5. Predictions of stress fibre and FA distributions

We consider convex- and concave-shaped ligand patterns on rigid substrates. A planar pattern is *convex* if it contains *all* the line segments connecting any pair of its points. The circle and right-angled triangle are examples. The V-, T-, Y- and U-shaped patterns studied by Théry *et al*. (2006) exemplify the concave patterns. To emphasize the influence of the shape, the dimensions of the cells and patterns have been chosen such that (i) they are consistent with those employed by Théry *et al*. (2006) and (ii) all cells have the same nominal area, approximately 900 μm^2^. In this two-dimensional analysis we assume that the cell has unit thickness, i.e. *b*=1 μm.

### 5.1 Convex patterns: stress-fibre-free initial state

*A circular ligand patch*, radius *R*=17.5 μm, is covered completely by a two-dimensional circular cell having equal radius in its undeformed configuration. The cell is initially stress and stress-fibre free $\left(\Sigma_{ij}=\eta (\phi )=0\right)$ with spatially uniform low- and high-affinity integrin concentrations, given by equations (2.12*a*) and (2.12*b*) with *Φ*=*F*_*i*_=0. Contractility is initiated by the activation signal at time *t*=0, resulting in the build-up of stress fibres and the development of FAs. All calculations are for a single activation signal.

Since the cell with these boundary and initial conditions possesses circular symmetry, it is sufficient to consider only the spatial distributions of parameters along any radial direction. Spatial distributions of *Γ*, *ξ*_H_/*ξ*_0_ and *ξ*_L_/*ξ*_0_ along the radius *r* are plotted in figure 3 at five selected times. Note that the FAs, parametrized by *ξ*_H_/*ξ*_0_, form very early in the process while the stress-fibre structure evolves over a much longer time scale. At *t*=0, concentrations *ξ*_L_/*ξ*_0_ and *ξ*_H_/*ξ*_0_ are spatially uniform with *ξ*_L_/*ξ*_H_∼150. The activation signal (applied at *t*=0) initiates the formation of stress fibres and their contractility, resulting in the ensuing sequence of events.

*Step I*. Mechanical equilibrium dictates that the newly formed stress fibres apply high tractions near the cell edge, at *r*/*R*≈1.

*Step II*. These high tractions reduce the chemical potentials of the high-affinity integrins (equation (2.9)) along the cell edge.

*Step III*. In order to maintain thermodynamic equilibrium, low-affinity binders transform to their high-affinity states at sites of high traction.

*Step IV*. The cytoskeleton and its associated FAs gradually evolve under these conditions until a steady state is reached.

In the model, the concentration of the low-affinity integrins on the membrane does not change significantly after time *t*/*θ*=9.1 (figure 3*c*) because diffusion has been neglected. This choice has been made based on more comprehensive calculations (Deshpande *et al*. in press) which demonstrate that the concentration of the low-affinity binders equalizes on a time scale much longer than that for the processes depicted in figure 3. This happens without modification of the stress-fibre distribution, the associated stresses, or the high-affinity integrins. It follows that the circular variance measure, *Γ*, as well as the distribution of high-affinity integrins, *ξ*_H_/*ξ*_0_ (figure 3 at time *t*/*θ*=9.1) are representative of the steady-state condition of the cell, whereupon the free energy has been minimized and homogenized.

Note that *Γ*, depicted in figure 3*a*, is a measure of the circular variance, with *Γ*≈1 when the stress fibres are highly aligned and *Γ*≈0 for an isotropic distribution. In the cell interior an almost isotropic network is developed (and hence *Γ*≈0), while at the cell periphery the fibres form preferentially along the cell edge, giving high values of *Γ*. The high-affinity integrins cluster by saturation of their concentration at the edge of the cell that then proceeds inwards to grow a FA around the perimeter (figure 3*b*). This phenomenon agrees with the experimental observation that FAs grow towards the direction from which the stress fibres apply traction (Riveline *et al*. 2001). However, symmetry breaking behaviour in the growth of FAs is not inherent in the model and needs to be incorporated in a manner that will be demonstrated in §5.2.

The coupling between the stress fibre and FA formation can be visualized by cross plotting the concentration of high-affinity integrins (representing the FAs) and the circular variance (representing the stress fibres) at two spatial locations (figure 4). In figure 4, the time after the signal has been imposed is implicit and the value of the normalized time *t*/*θ* is indicated at selected points on the plots (observe that *Γ* in these cases increases monotonically with time). The plot closer to the periphery (*r*/*R*=0.95, figure 4*a*) indicates that the integrin and stress-fibre concentrations both increase monotonically and reach an effective ‘endpoint’ when steady state is attained for *t*/*θ*>15 with nearly all integrins in their high-affinity state (*ξ*_H_/*ξ*_0_∼1) and a corresponding stress-fibre concentration, *Γ*≈0.2. Further from the periphery (at *r*/*R*=0.9, figure 4*b*), the integrin concentration attains a maximum while the stress-fibre concentration is still increasing. At the endpoint corresponding to the steady state only half of the integrins are in their high-affinity state and the stress-fibre concentration is much diminished (*Γ*≈0.08). This trend continues at yet smaller values of *r*/*R*. The results in figure 4*b* indicate that the force imposed by stress fibres on the high-affinity integrins increases to a maximum at time *t*/*θ*=0.72 and falls thereafter. Inspection of figure 3*b* suggests that this behaviour is associated with the development of FAs along the perimeter, causing the tractions near the cell edge to become progressively more localized, a feature that sets in at *t*/*θ*≈0.72.

*Next a cell on a triangular ligand pattern* is simulated (figure 5*a*) with dimensions larger than those employed by Théry *et al*. (2006) to retain a cell area comparable to that for the other shapes analysed. The steady-state distributions of *Γ* and *ξ*_H_/*ξ*_0_ are plotted in figure 5*b*,*c*. The processes affecting the cytoskeleton and FAs at earlier times are as described above in *steps I–IV* for a cell on a circular ligand patch. These *steps* rationalize the patterns shown in figure 5*b*,*c* and their descriptions will not be repeated. Consistent with observations (Chen *et al*. 2003; Théry *et al*. 2006), the calculations predict: (i) high FA concentrations along the cell periphery (figure 5*c*), (ii) high stress-fibre concentrations (as parametrized by *Γ*) along the cell periphery, with the fibres parallel to the edge (figure 5*b*), and (iii) lower *Γ* near the corners. In the interior an almost isotropic network of fine actin filaments is developed and hence *Γ*≈0. By contrast, adjacent to the periphery stress fibres form preferentially along the cell edge resulting in higher *Γ*. However, at the rounded corners, stress fibres are less aligned, resulting in lower *Γ* than along the edges. These results suggest that *ξ*_H_/*ξ*_0_ correlates well with the FA concentrations observed while the stress-fibre distributions are best characterized by *Γ*.

### 5.2 Convex patterns: effect of an initial anisotropic stress-fibre distribution

In a variety of situations, there is increased *persistence* of stress-fibre cables, i.e. there is a tendency for stress fibres to preferentially form and exist in a particular direction even though no external anisotropic stimuli are present (Andrä *et al*. 1998; Adam *et al*. 2000). Here we investigate whether the present model can capture anisotropic distributions in the absence of an anisotropic chemical or mechanical stimulus. For this purpose we again use circular cells, radius *R*=17.5 μm, on a circular ligand patch having equal area. The calculations are performed in two steps.

*Step 1*. Equation (2.2) was modified to generate an anisotropic activation signal such that

(5.1) $$C(\phi )=\{\begin{array}{ll} \text{exp}\;(-t_{\text{I}}/\theta ) & |\phi |\leq \phi_{\text{p}}\\ 0 & \text{otherwise} \end{array}.$$

This activation signal was applied to the stress and stress-fibre-free cell (with the choice *ϕ*_p_=10°) and the cell allowed to attain a steady state. As parametrized by *Γ* (figure 6*a*), the stress fibres form preferentially in the *ϕ*≈0° direction.

*Step 2*. An isotropic activation signal is applied to this anisotropic stress-fibre structure. The corresponding distributions of *Γ* after attaining steady state (figure 6*b*) reveal that, even towards the cell interior, an aligned stress-fibre structure persists with no distinct concentration of aligned fibres near the periphery.

We performed a control calculation with the same parameters except that both signals were isotropic. The steady-state distributions of *Γ* after the second signal (figure 6*c*) now reveal a configuration having circular symmetry with *Γ*≈0 towards the interior and high along the periphery. The corresponding steady-state FA distributions are included in figure 7*a*,*b*. While the distributions are similar in both cases (with the adhesions concentrating along the periphery and preserving the circular symmetry), the concentrations are higher in the control case. These results suggest that an anisotropic stress-fibre distribution does not necessarily give rise to anisotropic FA distributions. Conversely, an anisotropic adhesion pattern is not a requirement to generate an anisotropic stress-fibre distribution.

### 5.3 Concave patterns: stress-fibre-free initial state

The precise dimensions of the patterns analysed are sketched in figure 8 along with the undeformed cell shapes associated with each. We assume that only the initially stress and stress-fibre-free cell can adhere to the substrate on the ligand pattern. At *t*=0 the concentrations of the integrins on that portion of the cell in contact with the ligand patch are given by equations (2.12*a*) and (2.12*b*) with *Φ*=*F*_*i*_=0. The integrin concentrations on those portions of the cell not in contact with the ligand patch are specified by equations (2.15*a*) and (2.15*b*). All calculations employ a single isotropic activation signal (equation (2.2)) applied at *t*=0.

The time evolution of *Γ* and *ξ*_H_/*ξ*_0_ for the V-shaped pattern are shown in figures 9 and 10, respectively. The processes involved are the same as those described in §5.1 and denoted as *steps I–IV*. With increasing time, FAs (as parametrized by *ξ*_H_/*ξ*_0_) develop along the periphery of the ligand patch while highly aligned stress fibres (as measured by the quantity *Γ*) form preferentially along the non-adhered edge. In the simulations, the FA distribution attains a steady state at *t*/*θ*≈2 while the actin cytoskeleton arrangement stabilizes by *t*/*θ*≈7.

The FAs at the perimeter of the ligand patch locally anchor the cell to the underlying substrate. In the absence of adhesions, points in the cell are free to move. Accordingly, the non-adhered edges of the cell, lying outside the ligand patch, displace under the effect of stress-fibre contractility. The dominant orientation of the stress fibres along the non-adhered edge is that parallel to the perimeter: albeit that, prior to steady state, a small concentration of stress fibres form orthogonal to the edges. Since the traction-free boundary condition requires the tension to be zero in the stress fibres orthogonal to the non-adhered edges, they shrink at a strain rate given by $-\eta {\dot{\epsilon }}_{0}/{\bar{k}}_{\text{v}}$; see equation (2.4). This shrinkage pulls the non-adhered edges towards the interior of the cell to form a concave arc.

To characterize this effect we define a radius of curvature of the non-adhered edges as follows. The ends are defined in the undeformed configuration as those points where the non-adhered edges first make contact with the ligand patch. These points are marked in figure 8*a* for the V-shaped patch using crosses. The chord joining these ends has length *ℓ*=32.5 μm. This length, along with the perpendicular distance *w* of the non-adhered edges' mid point to this chord (i.e. the sagitta), defines the radius of curvature as

(5.2) $$\lambda =\frac{{\left(\ell /2\right)}^{2}+w^{2}}{2w}.$$

The predictions of the variation of *λ* with time are plotted in figure 11. Since the edges are straight at time *t*=0 the curvature is initially infinite (*λ*=∞). As the cell contracts, the curvature increases with a steady state attained at *t*/*θ*≈7. The variations of *λ* with time (for ${\bar{k}}_{\text{v}}=7$ and 10, in addition to the reference ${\bar{k}}_{\text{v}}=6$) are included in figure 11. It will be shown subsequently (figure 15) that stresses along the non-adhered edges are rather small. Thus, when the stress fibres orthogonal to the free edge have non-zero concentration, they contract at a strain rate ${\dot{\epsilon }}_{\text{U}}\approx -\eta {\dot{\epsilon }}_{0}/{\bar{k}}_{\text{v}}$ so as to maintain the traction-free boundary conditions on the non-adhered edge. This contraction of the fibres results in the curvature of the non-adhered edges: with decreasing ${\bar{k}}_{\text{v}}$, the contraction rate increases, thereby increasing the curvature.

The predicted steady-state actin and vinculin distributions for the V-, T-, Y- and U-shaped patterns, characterized by the parameters *Γ* and $\xi_{\text{H}}/\xi_{0}$, respectively (figures 12 and 13), are amenable to direct comparison with the experimental images of Théry *et al*. (2006; figure 1: the experimental images represent the average distributions over 10 nominally identical cells). Remarkable consistency is apparent, with the model capturing the following essential features: (i) the stress-fibre concentrations *Γ* are the highest along the non-adhered cell edges, (ii) *Γ* is lowest near the interior of the ligand patch and increases towards the edges, and (iii) the vinculin or FA concentrations are highest along the edges. These predictions are rationalized as follows:

- similar to the convex shapes, the highest tractions are developed at the edges of the ligand patch, resulting in the strongest FAs at those locations,
- nearly isotropic stress-fibre networks form in the interior, resulting in *Γ*≈0, and
- near the non-adhered edge, stress fibres mainly develop parallel to the edge which gives rise to the high values of *Γ* in those locations.

The simulated distributions of stress fibres (figure 12) conform satisfactorily with the experiments (figure 1). But convergence is less reliable for the vinculin distribution (figure 13) particularly the discrepancy in the relative concentrations on the inner and outer edges. More significantly, the simulations predict a uniform patch of FAs along the edge of the ligand patch while the experiments infer an elevation in the concentrations at locations of high curvature. A possible interpretation is suggested by the time evolution of *ξ*_H_/*ξ*_0_ (figure 10). Namely, since FAs first assemble at the high curvature segments of the ligand patch, as observed in the experiments (figure 1), perhaps the images pertain to times preceding steady state. Alternatively, continued assessment may reveal that refinements to the model are needed to resolve the discrepancies.

A comparison between the measured and predicted steady-state radii of curvature *λ*_ss_ for each concave shape is included in figure 14*a*. The results are presented for the reference ${\bar{k}}_{\text{v}}$, chosen to ensure correspondence for the V-shaped patch. Reassuringly, good agreement between the predictions and measurements is obtained for all shapes *with the same set of parameters*. Moreover, consistent with the observations of Théry *et al*. (2006) the curvatures decrease with increasing number of non-adhered edges. Note that, in the images in figure 1, there is uncertainty regarding the positions of the endpoints of the non-adhered cell edges and of the locations of maximum displacement: given this uncertainty, the 20% discrepancy between predictions and observations is considered acceptable. The effect of ${\bar{k}}_{\text{v}}$ on the predictions is shown in figure 14*b*. The curvature increases (*λ*_ss_ decreases) with decreasing ${\bar{k}}_{\text{v}}$ for all the concave shapes.

Given the consistency between the measurements and predictions of the actin and vinculin distributions and the curvatures of the non-adhered edges, we employ the model to estimate the steady-state distributions of the normalized maximum principal stresses, *σ*_p_/*σ*_max_ (figure 15). Line segments indicating the directions of the principal stresses are included with length proportional to *Γ*. In contrast to the *Γ* distributions (figure 12), we observe that the maximum stresses develop towards the interior of the ligand patches with the stresses rather small near the non-adhered edges. However, the principal stress directions are parallel to the cell edges along the non-adhered edges.

The response of cells in experiments on micro-patterned substrates involves two distinct stages (Théry *et al*. 2006): (i) cell spreading over the ligand patch and (ii) reinforcement and contractility resulting in the formation of the FA and stress-fibre structures. In the foregoing modelling exercise, only stage (ii) has been analysed by assuming an initial spread state of the cell. The coupling of cell spreading and contractility remains a challenge for future research. Another key observation is that the concentration of FAs is reduced for cells on compliant substrates (Saez *et al*. 2005; Yeung *et al*. 2005). The *coupled* stress fibre and FA model presented is expected to reproduce this observation. Recall that a key feature of the cytoskeletal model is that it will predict a reduced concentration of stress fibres (and associated forces exerted by the cell) for cells on compliant substrates (Deshpande *et al*. 2006). With reduced forces *F*_*i*_ in equation (2.9), there will be a smaller tendency for low-affinity integrins to transform to high-affinity integrins in order to maintain thermodynamic equilibrium giving rise to a smaller concentration of FAs for cells on compliant substrates. Simulations to quantify this effect are beyond the scope of this paper and will form the basis for future studies.

## 6. Concluding remarks

The response of cells on substrates imprinted with ligand micro-patterns is investigated by employing a two-dimensional bio-chemo-mechanical model that couples the remodelling of the actin/myosin stress fibres in the cytoskeleton with the formation and growth of FAs. The response of cells on two classes of ligand patterns is investigated: (i) convex-shaped circular and triangular patterns and (ii) V-, T-, Y- and U-shaped concave patterns. The primary difference between these two shapes is that cells on the concave-shaped patterns have non-adhered edges while all cells' edges are adhered on the convex patterns. With the experimental actin images interpreted as measuring the circular variance of the actin stress fibres, the model accurately captures key experimental observations including: (i) the development of high stress fibre and FA concentrations at the periphery of circular and triangular, convex-shaped ligand patterns, (ii) the formation of high FA concentrations along the edges of the V-, T-, Y- and U-shaped concave ligand patterns, and (iii) the development of aligned stress fibres along the non-adhered edges of cells on the concave ligand patterns. When appropriately calibrated, the model also accurately predicts the radii of curvatures of the non-adhered edges of cells on the concave ligand patterns. Thus, we have demonstrated that this rather simple model captures the observations with remarkable accuracy even though it does not incorporate details of the many complex signalling pathways, and in the present treatment we have modelled the cells as two dimensional.

Previously, this model has been used to explain such observations as: (i) the scaling of the forces exerted by cells on a bed of micro-needles (Deshpande *et al*. 2007), (ii) the influence of cell shape and boundary conditions on the development of structural anisotropy (Deshpande *et al*. 2006), (iii) the high concentration of stress fibres at FAs (Deshpande *et al*. 2006, 2007), and (iv) the formation of stress fibres perpendicular to the direction of cyclic stretching (Wei *et al*. in press). We have shown that the coupled contractility and FA model includes the ability to ‘sense’ mechanical and chemical heterogeneity in the cell environment. *In vivo* the ability of cells to detect such heterogeneities is fundamental for tissue homoeostasis. Moreover, there is a connection between the stresses at the cell periphery calculated within the model and wound closure.

Taken together, these findings suggest that this framework represents a step towards developing a more complete model for the coupled bio-chemo-mechanical responses of single cells and ensembles of cells. Although our comparisons with various cell phenomena have been restricted to conditions *in vitro*, we believe that the model is valid for cells *in vivo* and may be capable of simulating the response of a cell interacting with surroundings characterized by their mechanical properties, such as elasticity or viscoelasticity. Prior to tackling that challenge, the capabilities and implementation of our model will be extended to enable three-dimensional simulations, and to include more realistic models for signalling. Other cellular phenomena, such as migration and proliferation, will require substantial developments of the model.

## Figures and Tables

**Figure 1:**
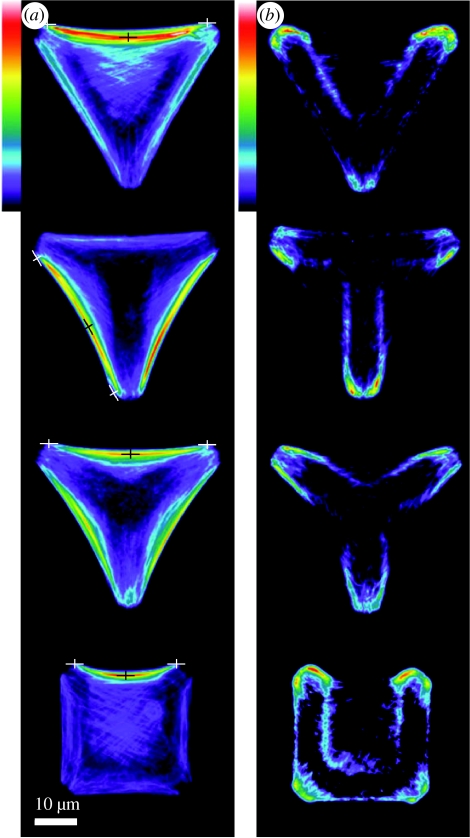
Experimental images of average (*a*) actin and (*b*) vinculin distributions for hTERT-RPE1 cells plated on V-, T-, Y- and U-shaped ligand micro-patterns. The crosses in (*a*) indicate the endpoints and location of maximum displacement of the non-adhered cell edges used in estimating the radii of curvatures of those cell edges. The length of the triangular edge is 46 μm and a scale bar is included for reference purposes. Reproduced with some modifications from Théry *et al*. (2006). Copyright © John Wiley & Sons, Ltd.

**Figure 2:**
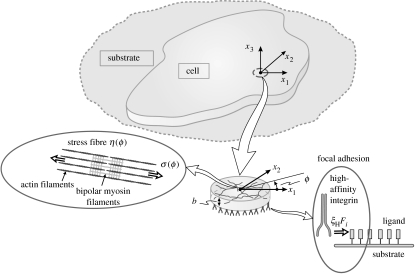
Schematic of a cell on a ligand-coated substrate. The network of stress fibres and the integrin–ligand complex part of the FA are shown as insets.

**Figure 3:**
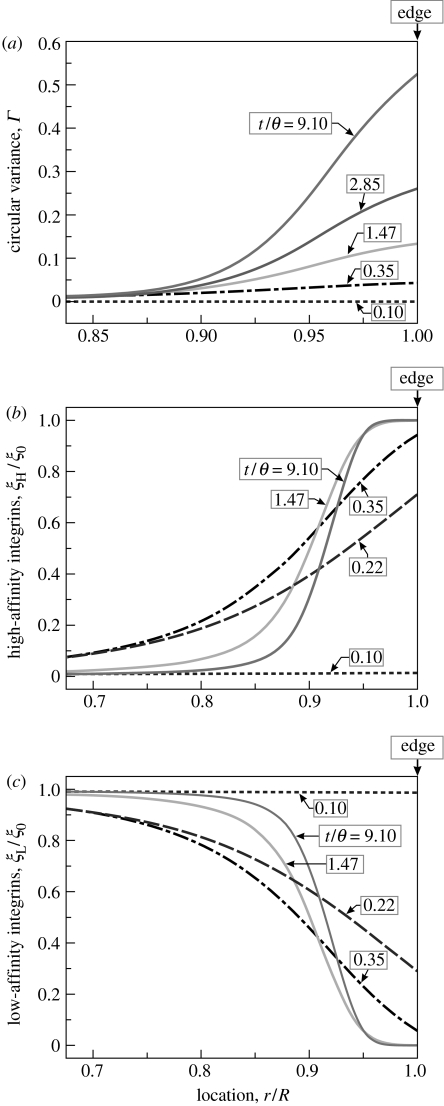
Predictions of the distributions of the stress-fibre and integrin concentrations along the radius of a circular cell of radius *R*=17.5 μm on a circular ligand patch of equal radius. The distributions are shown at five selected times. In (*a*) the stress-fibre concentrations as parametrized by *Γ* are plotted while (*b*,*c*) show the normalized high, *ξ*_H_/*ξ*_0_, and low, *ξ*_L_/*ξ*_0_, affinity integrin concentrations, respectively.

**Figure 4:**
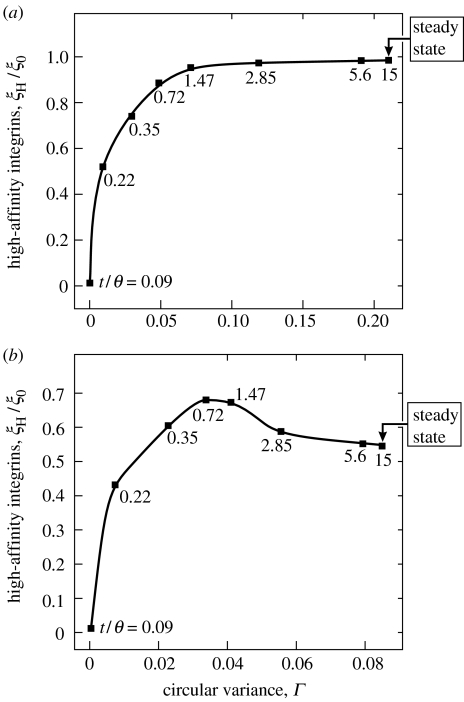
Predictions of the relationship between the stress-fibre concentration, *Γ*, and the high-affinity integrin concentration, *ξ*_H_/*ξ*_0_, at two locations, *r*, near the periphery of the circular cell of radius *R*=17.5 μm on a circular ligand patch of equal radius: (*a*) *r*/*R*=0.95 and (*b*) *r*/*R*=0.9. The parameter *Γ* increases monotonically with time and the selected normalized times *t*/*θ* are marked adjacent to the square symbols.

**Figure 5:**
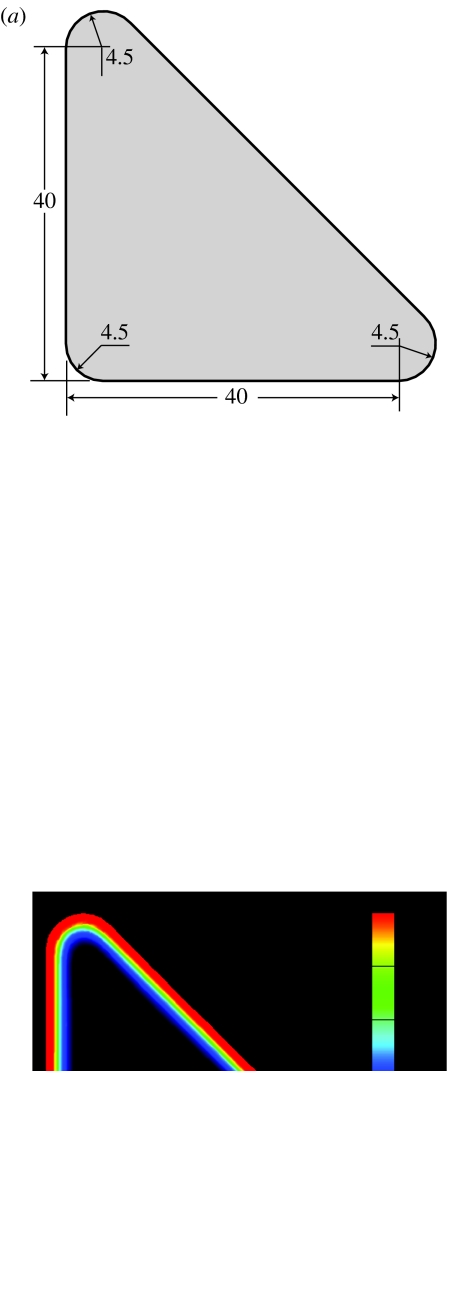
Predictions of the steady-state distributions of the stress-fibre and integrin concentrations for a triangular cell on a ligand pattern of the same shape. (*a*) Sketch of triangular pattern (all dimensions are in micrometres). Steady-state distributions of (*b*) *Γ* and (*c*) *ξ*_H_/*ξ*_0_. In (*b*) the direction *ϕ*_S_ is indicated by line segments having length scaled by *Γ*.

**Figure 6:**
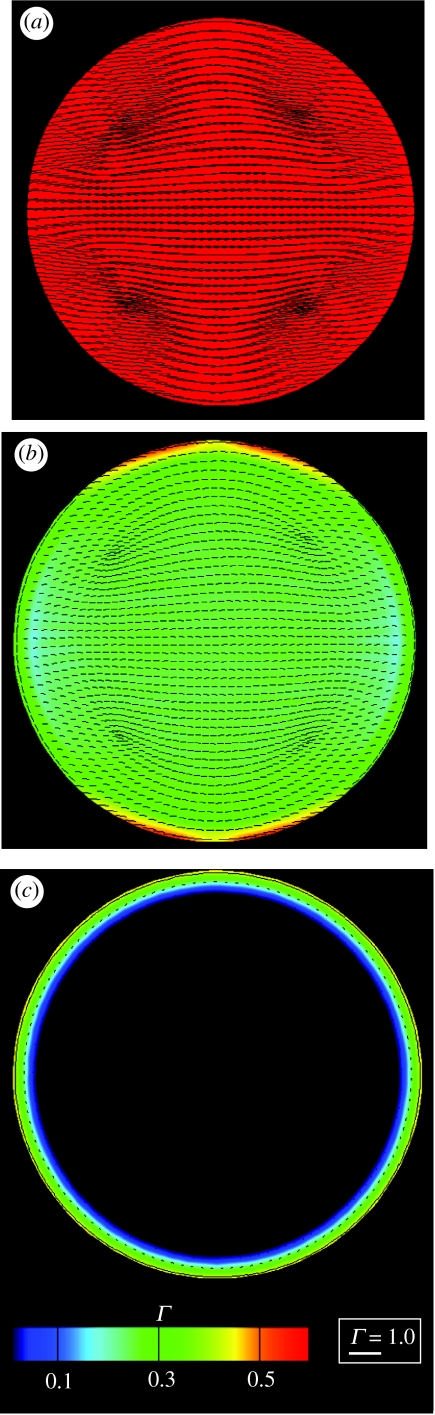
Predictions of the stress-fibre distributions in a circular cell of radius *R*=17.5 μm on a circular ligand patch of equal radius. Steady-state distributions of *Γ* (*a*) after application of an anisotropic activation signal that constrains stress-fibre formation to |ϕ|≤10° and (*b*) after a second isotropic activation signal is applied to the cell with the initially anisotropic stress-fibre distribution shown in (*a*). (*c*) The control case of the steady-state distributions of *Γ* after the application of two isotropic activation signals. The direction *ϕ*_S_ is indicated by line segments having length scaled by *Γ* in all cases.

**Figure 7:**
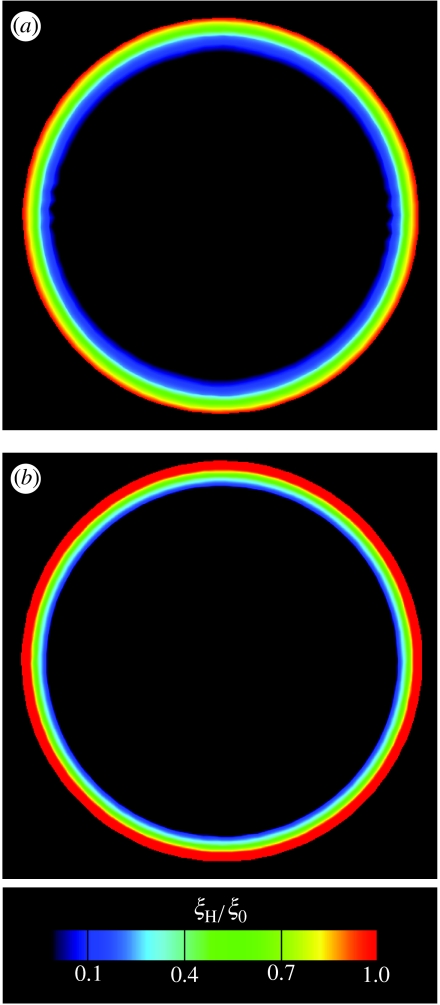
Predictions of the steady-state FA distributions as parametrized by *ξ*_H_/*ξ*_0_ in a circular cell of radius *R*=17.5 μm. The FA distributions in (*a*) and (*b*) correspond to the stress-fibre distributions in figure 6*b* and *c*, respectively.

**Figure 8:**
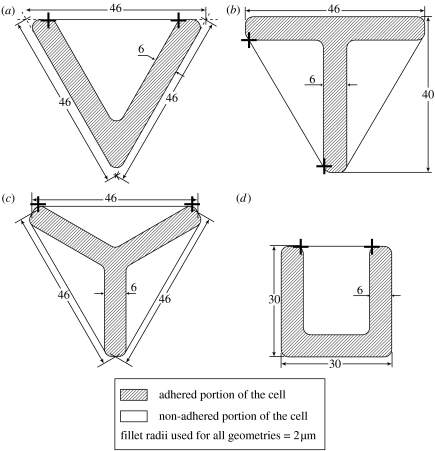
Sketches of ligand pattern and cell shape for the (*a*) V-, (*b*) T-, (*c*) Y- and (*d*) U-shaped ligand patterns. The endpoints of the non-adhered cell edges are marked by crosses and all dimensions are in micrometres.

**Figure 9:**
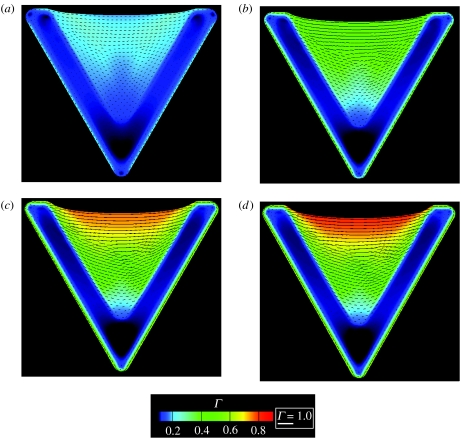
Predictions of the distributions of the stress fibres as parametrized by *Γ* for a cell on the V-shaped ligand pattern. The predictions are shown at four selected times (*a*, *t*/*θ*=2.1; *b*, *t*/*θ*=3.97; *c*, *t*/*θ*=7.85; *d*, *t*/*θ*=15) and the direction *ϕ*_S_ is indicated by line segments whose length is scaled by *Γ* in all cases.

**Figure 10:**
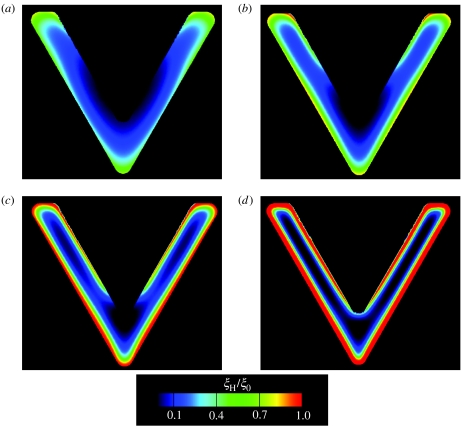
Predictions of the distributions of vinculin or FA concentration as parametrized by *ξ*_H_/*ξ*_0_ for a cell on the V-shaped ligand pattern. The predictions are shown at four selected times (*a*, *t*/*θ*=0.22; *b*, *t*/*θ*=0.35; *c*, *t*/*θ*=0.72; *d*, *t*/*θ*=2.1).

**Figure 11:**
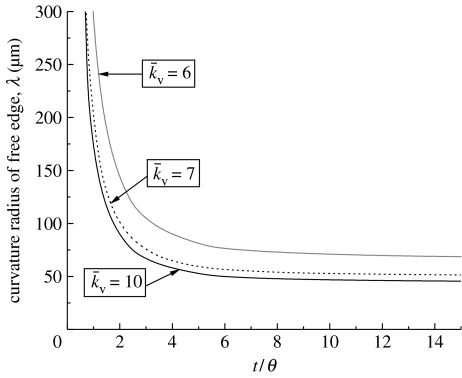
Predictions of the time evolution of the radius of curvature, *λ*, of the non-adhered edge of the cell on the V-shaped ligand pattern. Predictions are shown for three values of ${\bar{k}}_{\text{v}}$ (including the reference value, ${\bar{k}}_{\text{v}}=6$).

**Figure 12:**
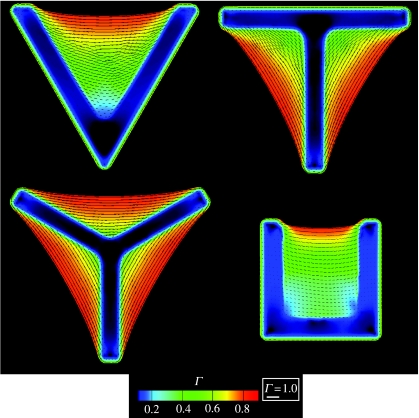
Predictions of the steady-state distribution of actin as parametrized by *Γ* for the four concave ligand patterns investigated here and sketched in figure 8. The direction *ϕ*_S_ is indicated by line segments whose length is scaled by *Γ* in all cases.

**Figure 13:**
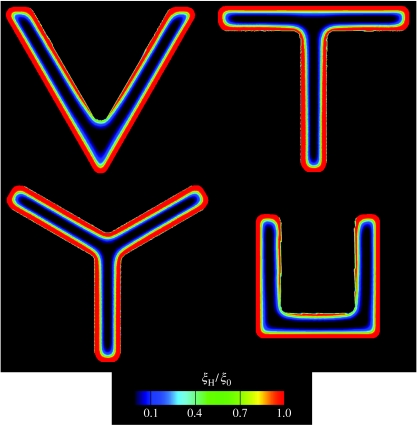
Predictions of the steady-state distribution of vinculin as parametrized by *ξ*_H_/*ξ*_0_ for the four concave ligand patterns investigated here and sketched in figure 8.

**Figure 14:**
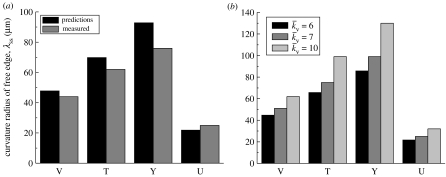
(*a*) A comparison of the measured and predicted steady-state radii of curvature, *λ*_ss_, of the non-adhered edges of the cells on four concave ligand patterns. The predictions employ the reference value, ${\bar{k}}_{\text{v}}=6$. (*b*) The predictions of *λ*_ss_ for three selected values of ${\bar{k}}_{\text{v}}$ including the reference value.

**Figure 15:**
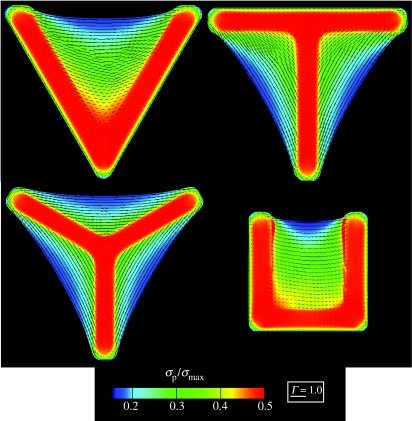
The steady-state distributions of the normalized maximum principal stress, *σ*_p_/*σ*_max_, in the cells on the four concave ligand patterns. The direction of the maximum principal stress is indicated by line segments whose length is scaled by *Γ*.

## Footnotes

While for simplicity the signal, *C*, may be thought of as the normalized concentration of Ca^2+^ we realize also that the signalling process is much more complicated than a simple flood of calcium ions, involving as it does a cascade of reactions implicating many different G-proteins. Our more complete description of the biochemistry underlying our model, presented in Deshpande *et al*. (2007) and its online supporting material, recognizes this point, including the implications for signal decay.

Note that in our model these elastic properties do not represent the full mechanical response of a live cell when it is distorted by a measurement device such as an AFM tip. In our interpretation of such experiments carried out on live cells, the triggering of signalling stimulated by the measurement device, and the consequent remodelling and contractility of the cytoskeleton, will give rise to a complex response in which some of the forces measured in reaction to the displacements imposed by the measuring device will be due to active stress-fibre contractility. Thus, the apparent elastic modulus calculated from such experiments will be significantly higher than the value of 0.08 kPa used as our passive Young's modulus.
